# Likely Female-to-Female Sexual Transmission of HIV — Texas, 2012

**Published:** 2014-03-14

**Authors:** Shirley K. Chan, Lupita R. Thornton, Karen J. Chronister, Jeffrey Meyer, Marcia Wolverton, Cynthia K. Johnson, Raouf R. Arafat, M. Patricia Joyce, William M. Switzer, Walid Heneine, Anupama Shankar, Timothy Granade, S. Michele Owen, Patrick Sprinkle, Vickie Sullivan

**Affiliations:** 1 Houston Department of Health and Human Services, Houston, Texas; 2 Division of HIV and AIDS Prevention, National Center for HIV, Hepatitis, STD, and TB Prevention, CDC

In August 2012, the Houston Department of Health contacted CDC regarding the rare transmission of human immunodeficiency virus (HIV) likely by sexual contact between two women. The case was investigated, and laboratory testing confirmed that the woman with newly diagnosed HIV infection had a virus virtually identical to that of her female partner, who was diagnosed previously with HIV and who had stopped receiving antiretroviral treatment in 2010. This report describes this case of HIV infection, likely acquired by female-to-female sexual transmission during the 6-month monogamous relationship of the HIV-discordant couple (one negative, one positive). The woman with newly acquired infection did not report any other recognized risk factors for HIV infection, and the viruses infecting the two women had ≥98% sequence identity in three genes. The couple had not received any preventive counseling before acquisition of the virus by the woman who had tested negative for HIV. HIV-discordant couples should receive counseling regarding safer sex practices, and HIV-infected partners should be linked to and retained in medical care.

Transmission of HIV between women who have sex with women (WSW) has been reported rarely and is difficult to ascertain. The potential for HIV transmission by female-to-female sexual contact includes unprotected exposure to vaginal or other body fluids and to blood from menstruation, or to exposure to blood from trauma during rough sex. Other potential exposures associated with HIV transmission in WSW that must be ruled out include injection drug use (IDU), heterosexual sex, tattooing, acupuncture, piercing, use of shared sex toys between the partners and other persons, exposure to body fluids of others, and receipt of transplants or transfusion.

## Epidemiologic Findings

The woman who acquired HIV was aged 46 years and had a history of heterosexual intercourse, but not in the 10 years before HIV infection. She reported three female sexual partners in the preceding 5 years but said she had no IDU, receipt of tattoos, acupuncture, transfusions, transplants, or any other recognized HIV risk behavior. The woman supplemented her income by selling her plasma and had tested negative for HIV by HIV-1/2 enzyme immunoassay (EIA) serology screening after donating plasma in March 2012.

In April, 10 days after donating plasma, the woman went to an emergency department with a sore throat, fever, vomiting, decreased appetite, pain on swallowing, dry cough, frequent diarrhea, and muscle cramps. At that time, she was again tested for HIV by EIA serology screening, and the results were negative. She was treated with azithromycin for a presumed upper respiratory infection and discharged. Eighteen days later, the woman attempted to sell plasma but was refused because she tested positive for HIV by EIA serology screening followed by an HIV-1 Western blot test. On July 5, results of repeated EIA and Western blot tests conducted on the woman at a health clinic were positive for HIV infection.

The likely source of the patient’s new HIV infection was her female sex partner aged 43 years who had tested positive for HIV in September 2008 when she had an HIV-1 viral load of 82,000 copies/mL and a CD4+ T-lymphocyte count of 372 cells/mm^3^ (25%). The partner began antiretroviral treatment in February 2009 but stopped in November 2010. Although she had esophageal candidiasis and weight loss at the time of her HIV diagnosis, her HIV-1 viral load had decreased to 178 copies/mL, and her CD4+ T-lymphocyte count had increased to 554 cells/mm^3^ (44%) by January 2011, when she was lost to follow-up.

The couple reported routinely having unprotected (using no barrier precautions) oral and vaginal contact and using insertive sex toys that were shared between them but were not shared with any other persons. They described their sexual contact as at times rough to the point of inducing bleeding in either woman. They also reported having unprotected sexual contact during the menses of either partner. The recently infected woman reported that her partner was her only sexual contact during the 6 months before her seroconversion.

## Phylogenetic Analyses

On September 10, 2012, the newly infected woman tested positive for HIV by HIV-1/2/O EIA, and her HIV-1 Western blot was positive for all bands. Her Multispot test was reactive to HIV-1 only, and she had an HIV-1 viral load of 23,600 copies/mL. The partner’s blood tested positive by HIV-1/2/O EIA, and her HIV-1 Western blot was positive for all bands. Her Multispot test was reactive to HIV-1 only, and she had a HIV-1 viral load of 69,000 copies/mL. HIV-1 polymerase (*pol*), group antigen (*gag*)*,* and envelope (*env*) sequences were amplified by polymerase chain reaction from specimens from both women. Phylogenetic analyses of the *pol* and *env* sequences revealed that both women had highly related sequences with pairwise nucleotide identity of 98.7% in *gag* and 98.0% in both *env* and *pol*. Neither *pol* sequence had any major drug resistance mutations but shared the following polymorphisms: protease (M36I, R41K, and L63T) and reverse transcriptase (R83K, K122E, I178L, and R211K).

### Editorial Note

This report describes a case of HIV transmission likely by sexual contact between female partners. Past confirmation of HIV transmission during female-to-female sexual contact has been difficult because other risk factors almost always are present or cannot be ruled out. In this case, other risk factors for HIV transmission were not reported by the newly infected woman, and the viruses infecting the two women were virtually identical.

Few previous reports describe HIV transmission between WSW. One case involved a woman diagnosed in the Philippines who reported sexual contact exclusively with women and said she did not use injection drugs; however, no source of transmission was confirmed (1). Another instance of HIV transmission between WSW was reported for a woman aged 20 years with no other risk behaviors who said she had a 2-year relationship and unprotected intercourse with a female partner known to be HIV-infected (2). The woman and her partner had identical HIV-1 drug resistance mutations, but no phylogenetic linkage testing was conducted.

More commonly, HIV infections in WSW have been attributed to risk behaviors such as IDU or to concomitant heterosexual sex. A study of 18 HIV-discordant WSW couples followed for 3–6 months found no evidence of transmission, leading the authors to suggest that no risk for HIV transmission might exist in exclusive WSW couples (3). The same authors described the cases of 11 HIV-positive WSW and found that 10 used injection drugs and two provided a history of sexual activity with both men and women (4). In a cohort of 511 women with a history of female-to-female sexual contact, 470 (92%) reported having sex with both men and women, and 41 (8%) were WSW only; 13 women were found to have HIV infection, but none were categorized as WSW only (5).

To document female-to-female sexual contact in women who were HIV-positive, a survey of 960,000 female blood donors was conducted; of 144 women who tested positive for HIV infection, 106 were interviewed. Of these 106 women, 102 were heterosexual, three had a history of sex with both men and women, one reported having had sex with a person with a history of IDU whose sex was not given, and three women had a history of IDU. None of the 106 women reported female-to-female sexual contact as their only risk behavior (6). In another large survey conducted during 1986–1989, a total of 1,014 female patients in a clinic were interviewed, and 101 (10%) reported female-to-female sexual contact. Of the 101 WSW, 90% provided a history of sex with both men and women, and 37% reported IDU history. All 13 women who tested HIV-positive and reported female-to-female sexual contact also provided a history of sexual contact with men, and 12 reported IDU history (7).

A series of reports by CDC authors did not confirm HIV transmission by female-to-female sexual contact alone. In a 1990 report, among 79 women who were HIV-positive and had female-to-female sexual contact history only, 75 also had a history of IDU, and the remaining four had received transfusions (8). In a 1992 report, a total of 18,199 women with acquired immunodeficiency syndrome (AIDS) from the period 1980–1991 were examined; 164 of these women provided a history of female-to-female sexual contact. Of the 164, a total of 152 (93%) provided an additional history of IDU, and 12 (7%) had received blood before 1985 (9). In a 1994 report, of 1,122 women found to be HIV-positive, 65 (5.8%) had a history of female-to-female sexual contact. Of the 65, a total of 55 (85%) also had a history of sexual contact with men; 28 of the women with a history of sexual contact with both men and women also reported IDU. Of the 10 remaining women with exclusive female-to-female sexual contact, eight reported IDU, one had received a blood transfusion, and one was reported as having no other identified risk behavior (10).

What is already known on this topic?

Cases of human immunodeficiency virus (HIV) infection transmitted by sexual contact between women who have sex with women are rare and difficult to ascertain. Other, more common, modes of transmission, such as injection drug use and heterosexual sex, usually are difficult to rule out. However, female-to-female transmission is possible because HIV can be found in vaginal fluid and menstrual blood.

What is added by this report?

In 2012, a woman who reported no heterosexual sex in the previous 10 years, injection drug use, or other recognized risk factors for HIV infection tested HIV-positive during a 6-month monogamous relationship with a female sexual partner who was HIV-positive and had stopped receiving antiretroviral treatment in 2010. Phylogenetic analysis of the HIV virus from the two women showed that the viruses were virtually identical.

What are the implications for public health practice?

Discordant couples of any sex should know their HIV status and receive education and counseling services, especially instruction in safer sex practices. Persons identified as HIV-infected should be linked to and retained in medical care. A suppressed HIV viral load can result in better health outcomes and reduce the possibility of transmitting HIV infection to partners

This report describes likely female-to-female transmission of HIV-1 supported by phylogenetic analysis in a WSW couple who had unprotected sex during a 6-month monogamous relationship. Although rare, HIV transmission between WSW can occur. All persons at risk for HIV, including all discordant couples, should receive information regarding the prevention of HIV and sexually transmitted infections to prevent the HIV-negative partner from acquiring the infection. Furthermore, all persons identified as infected with HIV should be linked to and retained in medical care. Control of HIV infection with suppression of viral load can result in better health outcomes and a reduced chance of transmitting HIV to partners.
